# Supplementary material for: Lactobacillus rhamnosus TR08 Improves Dyslipidemia in Mice Fed with a High Fat Diet by Regulating the Intestinal Microbiota, Reducing Systemic Inflammatory Response, and Promoting Sphingomholipid Metabolism
Source: Molecules. 2022 Oct 29;27(21):7357. doi: 10.3390/molecules27217357 (PMC9655260; doi:10.3390/molecules27217357)
Supplement: Supplementary file 1 [file molecules-27-07357-s001.zip › molecules-1981462-supplementary.pdf]

Supplementary materials

Table S1 The primers for qPCR of various genera of bacteria

Genera	Primers (5′-3)′
Escherichia	F: TGCCGTAACTTCGGGAGAAGGCA<br>R:TCAAGGACCAGTGTTCAGTGTC
Enterococcus	F: CCCTTATTGTTAGTTGCCATCATT<br>R: ACTCGTTGTACTTCCCATTGT
Bifidobacterium	F: CGGGTGAGTAATGCGTGACC<br>R: TGATAGGACGCGACCCCA
Bacteroides	F: AGCAGTAGGGAATCTTCCA<br>R: CAGGGCTACACATGGAG

Table S2 The primers of inflammation factors in mouse spleen by qRT-PCR

Genes	Primers (5′-3)′
Mun-*β-actin*	F: ATGACCCAAGCCGAGAAGG<br>R: CGGCCAAGTCTTAGAGTTGTTG
Mun-*IL-2*	F: ACACCTTTAATTGGTCAACACGA<br>R: CCTGCTACGTTCTCTACCTCT
Mun-*IFN-γ*	F: GAACTGGCAAAAGGATGGTGA<br>R: TGTGGGTTGTTGACCTCAAAC
Mun-*IL-4*	F: GGTCTCAACCCCCAGCTAGT<br>R: GCCGATGATCTCTCTCAAGTGAT
Mun-*IL-10*	F: GAAGCTCCCTCAGCGAGGACA<br>R: TTGGGCCAGTGAGTGAAAGGGG
